# Glyconanoparticles as tools to prevent antimicrobial resistance

**DOI:** 10.1007/s10719-021-09988-6

**Published:** 2021-03-17

**Authors:** Laura Morelli, Laura Polito, Barbara Richichi, Federica Compostella

**Affiliations:** 1 Department of Medical Biotechnology and Translational Medicine, University of Milan, Via Saldini 50, 20133 Milan, Italy; 2 National Research Council, CNR-SCITEC, Via G. Fantoli 16/15, 20138 Milan, Italy; 3 Department of Chemistry ‘Ugo Schiff’, University of Florence, Via della Lastruccia 13, 50019 Sesto Fiorentino, FI Italy

**Keywords:** Antibacterial infections, Vaccine platforms, Saccharide antigens, Glycans

## Abstract

The increased phenomenon of antimicrobial resistance and the slow pace of development of new antibiotics are at the base of a global health concern regarding microbial infections. Antibiotic resistance kills an estimated 700,000 people each year worldwide, and this number is expected to increase dramatically if efforts are not made to develop new drugs or alternative containment strategies. Increased vaccination coverage, improved sanitation or sustained implementation of infection control measures are among the possible areas of action. Indeed, vaccination is one of the most effective tools of preventing infections. Starting from 1970s polysaccharide-based vaccines against *Meningococcus*, *Pneumococcus* and *Haemophilus influenzae* type b have been licensed, and provided effective protection for population. However, the development of safe and effective vaccines for infectious diseases with broad coverage remains a major challenge in global public health. In this scenario, nanosystems are receiving attention as alternative delivery systems to improve vaccine efficacy and immunogenicity. In this report, we provide an overview of current applications of glyconanomaterials as alternative platforms in the development of new vaccine candidates. In particular, we will focus on nanoparticle platforms, used to induce the activation of the immune system through the multivalent-displacement of saccharide antigens.

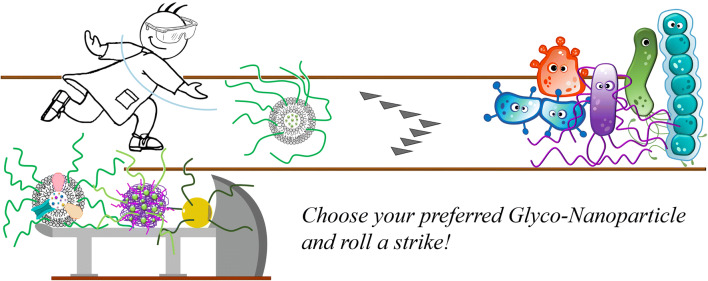

## Introduction

**Fig. 1:**
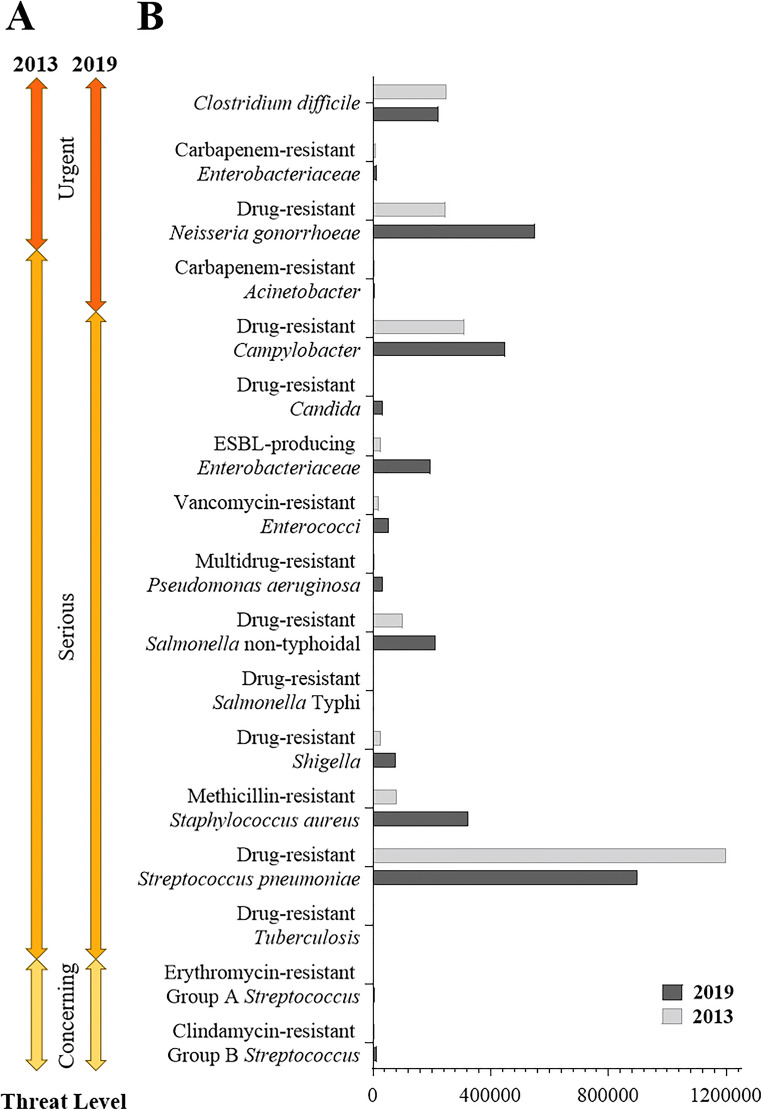
Estimated annual drug-resistant infections per year in US The figure, adapted from ref. [8], reports the US priority list of antibiotic resistant bacteria with the indication of the level of threat (**A**), and the number of estimated cases of resistant infections (**B**). The data are relative to the collection of data in 2019 and 2013

Microbial infections are one of the main threats that the global health system has to face to ensure a general control of citizens’ health. Millions of harmless microorganisms are cohabitants of humans, but some microbes pose a real danger for the worldwide community since they are the cause of many infectious diseases. Microorganisms enter the body via the epithelial surface and spread throughout the body via the circulatory system. The cells of the immune system, such as macrophages, phagocyte and eliminate these invading pathogens through defined killing pathways. However, some of them evade the macrophage digestion leading to intracellular infection, eventually resulting in disease [1]. Since the advent of penicillin in routine clinical use in the 1940s, thanks to increased production capacity, antibiotics have become the first line of defense to combat bacteria [2]. Early cases immediately revealed that the effect of the drug can be observed only if the administered doses are enough to complete the course of the medicine, underlining the importance of the informed use of antimicrobial agents. Since then, various classes or generations of antibiotics have been developed. Antibiotics are generally referred to as antibacterial and antifungal drugs, although in an all-comprehensive classification they are differentiated as antibacterial, antifungal and antiviral to reflect the group of microorganisms they antagonize [3]. Antibiotics can either kill or inhibit the growth of microorganisms by affecting their metabolism through inhibition of cell wall synthesis (penicillin and carbapenem), DNA synthesis (quinolones), or protein synthesis and enzyme activity (tetracyclines, sulfonamides and macrolides) [4]. Starting from 1990’s microbes have started to exhibit resistance against antimicrobials to ensure their survival. They have developed evolutionary defense mechanisms based on the chemical structure of the pharmacological agents directed at countering the effects of the antibiotic [5, 6]. Other factors contribute to increase the phenomenon of antimicrobial resistance (AMR), such as hospitalization for longer period of time in intensive care units with increment in the usage of medical enveloping devices and catheters, the administration of inadequate dosage of certain antibiotics, and the extensive use of antibiotics in agriculture [7]. The incidence of resistance varies widely among and within countries, and over time, but it has long been recognized worldwide as a major threat to human health with significant global economic and security implications. Figure 1 reports the number of infections estimated by the 2019 Antibiotic Resistance (AR) Threats Report in the United States for the key antibiotic-resistant germs that have to be considered as urgent, serious or concerning threats for human health [8]. The estimates are compared to the 2013 AR Threats report, to highlight that the risk has doubled or even tripled for some resistant pathogens like *Pseudomonas aeruginosa*, *Staphylococcus aureus*, *Neisseria gonorrhoeae*. Most of these pathogens are also listed in the Global Antimicrobial Resistance Surveillance System (GLASS) Report released in 2020 on high-priority pathogens that cause infections in humans worldwide [9]. Indeed, infections have in fact the potential to rapidly spread between countries due to the ever growing increase in human traveling and freight traffic. Early detection and prevention of these infections can have a large, positive impact on public health. The purpose of surveillance reports is to implement actions to improve the prevention of resistant infections that will continue to emerge worldwide and to give indications for a correct usage of antibiotics in all settings. Among the core actions proposed to fight microbial infections there is also a stimulus towards the development of vaccines.

Vaccination is one of the strongest and most effective weapons to decrease infection rates and at the same time contribute to reduce the excessive use of antibiotics and the evolution of resistant germs [10]. Vaccines work by causing the immune system to prevent the disease by targeting the presence of the pathogen in the body. The World Health Organization (WHO) campaign from 2000 to 2016 to promote a widespread use of the pneumococcal conjugate vaccine (PCV), which helped to protect against infection by *Streptococcus pneumoniae*, has significantly reduced the rate of death in children. More than 250,000 children were saved and had a new chance at life [8]. Extensive pneumococcal vaccination programs have substantially decreased the number of infections caused by vaccine strains and the spread of resistant *S. pneumoniae* strains [8]. Indeed, data reported in Fig. 1 for *S. pneumoniae* highlight that the level of threat in the US associated to the pathogen is declining after the introduction of routine vaccine campaign. In addition, antimicrobial resistance is higher in those countries where extensive vaccination has not yet been introduced [11]. Different generations of vaccines have been developed to elicit protective immunity against diseases: some are based on attenuated or killed whole organisms, others developed from pathogen subunits, and more recently also RNA or DNA based vaccines have been introduced [12].

Interestingly, the example of the vaccine against *Streptococcus pneumoniae* highlights the role of glycans as targets for the development of antibacterial therapeutics. Fragments of the polysaccharide capsules of the most virulent serotypes are included in pneumococcal vaccines preparations to stimulate a specific response of the host against the pathogen. Two different types of saccharide-based vaccines are in use: the polysaccharide vaccine, Pneumovax23, which includes purified preparations of pneumococcal capsular polysaccharides, not effective in infants, young adults, and immunocompromised people, and conjugate vaccines, like PCV-7 and PCV-10, where the saccharide fragments are conjugated to a carrier protein to elicit a more effective protection in all age groups. The conjugation to an immunogenic protein allows the switch from a T-independent response, typical of polysaccharide antigens, to a T-dependent one [13]. Glyco-vaccines support the notion that bacterial associated carbohydrates are optimal targets for the development of antimicrobial agents. The surface of bacterial cells is covered by a highly charged layer of membrane-bound biological macromolecules, named glycocalyx, which is composed of a network of polysaccharides and glycoproteins bearing acidic oligosaccharides. This layer functions as a barrier between the cell and its surrounding and protects the cell membrane from the direct action of physical forces and stresses allowing the membrane to maintain its integrity. The outermost part of the bacterial envelope of both Gram-negative and Gram-positive bacteria is represented by the capsule, composed of a polysaccharide structure specific for each bacterial serotype [14]. Several CPS fragment antigens have been tested in preclinical studies, or used in the formulation of licensed vaccines. More recently, other candidates for vaccine development could be identified among the other types of bacterial associated carbohydrates, such as the O-antigen portion of lipopolysaccharide (LPS) molecules in Gram-negative or cell wall-associated glycans in Gram-positive bacteria [15]. However, the vaccine pipeline is fragile because the process to develop a safe and effective vaccine is long and costly, and there are still no effective vaccines available against some of the most prevalent diseases including tuberculosis and malaria.

In this context, a recently emerged way to deliver virulence factors of the pathogen in the body could be offered by nanomaterials. The role of different types of nanomaterials as promising antimicrobial agents is already well established in other contexts [16]. For example, metal and metal oxide nanoparticles, in particular silver-based nanomaterial, have shown a promising antimicrobial activity; liposomes and polymeric nanoparticles are recognized as efficient biocompatible antimicrobial drug delivery systems, which allow sustained release of drug for a larger time period [17, 18]. More recently, the concept that nanosystems can be considered as efficient devices for the development of new generation vaccines has emerged. In this context, glyconanomaterials are defined as nanomaterials presenting functional carbohydrate entities at their surfaces. Glyconanomaterials have become useful platform for a range of applications, including vaccination where they are used as carriers of saccharide antigens. In addition, nanocarrier based delivery systems are endowed with good adjuvant properties, and offer the possibility to enhance vaccine antigen uptake thereby resulting in a stronger immune response. The antigen can be encapsulated within the nanoparticle or loaded on its surface. Sugar coated nanomaterials are characterized by a high carbohydrate surface density, and are able to mimic the carbohydrate arrangement at the cell surface through the multivalent presentation. Increased antigen stability to premature degradation, or a facilitated multivalent interaction with the surface receptors are key factors in providing a more efficient immune response comparable to soluble/unconjugated antigens [19].

In this minireview, we will focus on nanomaterials that are designed to induce a specific immune response against the carbohydrates that cover bacterial surface and represent the first point of contact with the host. To this end, we will discuss how the glycan-coating of nanoparticles can be harnessed to advance the development of new platforms for vaccine development. We will present our views on the types, preparation, and characterization of the nanoparticles used in this context. In particular, we will provide applicative examples of their use against select cases of bacteria representing urgent medical needs in AMR. We underline that, even though effective vaccination exists against e.g. *Streptococcus pneumoniae,* and some other selected pathogens do not represent a threat in terms of AMR, we will discuss them as interesting test cases for the application of glyconanotechnology in the framework of vaccine development.

## Nanomaterials for vaccine development: Methods for nanoparticle preparation and analysis

**Fig. 2:**
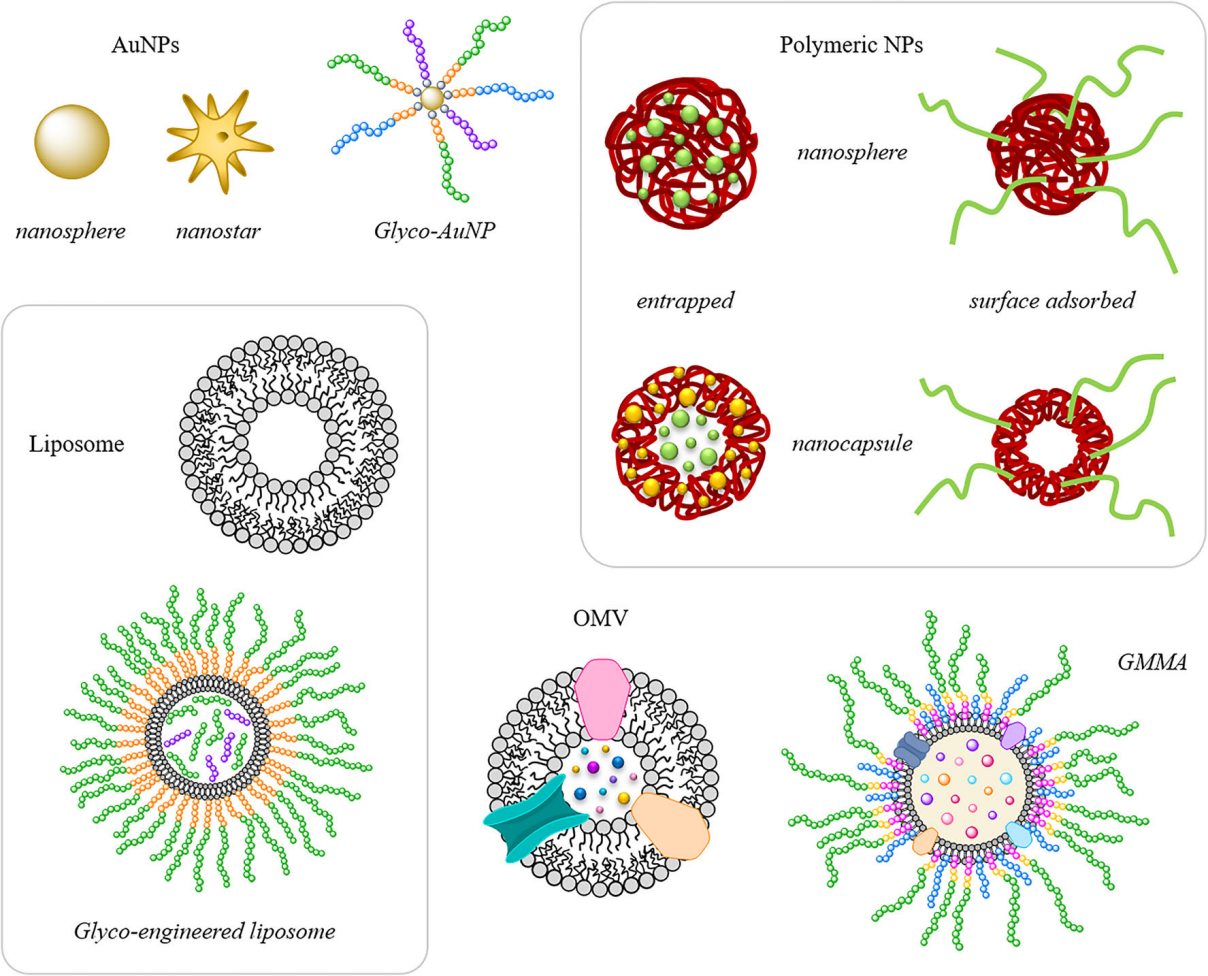
Nanosystems used for antigen presentation in vaccine development

Since the nature of nanomaterial plays a pivotal role in the design of a proper and effective glyconanoparticles (glycoNPs), a crucial step is the identification of highly biocompatible materials synthesized by robust and reliable protocols. Beside the type of core, the nanoparticle surface should be easily functionalized, allowing an accessible chemistry to graft carbohydrate payloads. An overview of the type of nanosystems that are used as carriers of saccharide antigens in nanoparticle vaccine development is herein outlined (Fig. 2).

Among the different materials, inorganic-based nanoparticles, mainly metals and metal oxides, are extremely attractive nanocarriers and, in the last years, have been exploited to develop new generation of glycoNPs [20–23]. In particular, gold nanoparticles (AuNPs) are extremely inert and bio-compatible nanomaterials but, despite their inertness, are characterized by a viable surface chemistry thanks to the strong soft–soft interaction that takes place between Au and sulfur [24]. AuNPs can be obtained by means of aqueous reduction of Au^III^ ions, allowing to fine-tune both size and core morphology. Following the well-known procedure developed by Turkevich in 1951 [25], it is possible to reduce HAuCl_4_ in hot water (100 °C) by sodium citrate, obtaining citrate-capped AuNPs in the range of 20–100 nm. Since the first publication, many modifications have been reported to increase the yield, the reproducibility and the monodispersion of the NP synthesis [24]. The subsequent process of surface functionalization with thiolated organic molecules takes place through a ligand exchange reaction during which the mild stabilization promoted by the carboxylic acid of citrate is substituted by the strong affinity of thiols toward gold. On the other hand, the synthesis of small and ultrasmall AuNPs, in the range of 2–8 nm can be achieved by means of another approach developed by Brust-Schiffrin which introduced the use of sodium borohydride (NaBH_4_) as stronger reductant [26]. Such conditions promote a fast nucleation of gold seeds which are immediately capped by the co-presence of proper thiols, affording stabilized surface capped AuNPs. Finally, it is worth noting the AuNPs unique optical properties which are based on the local surface plasmon resonance (LSPR) band [27, 28]. This phenomenon takes place when the electrons of the conductive band of a noble metal are confined in a quantum size and start to oscillate collectively when light imping on them. By UV-visible measurements it is possible to observe the typical plasmonic band centered at 520 nm for spherical 10 nm AuNPs. Nevertheless, LSPR band is strongly affected by the size, the shape, and the composition of plasmonic NPs representing a feature which can be tailored on the base of the final application [28, 29].

To characterize AuNP size and morphology the main techniques are the UV-visible light to evaluate the LSPR band, the dynamic light scattering (DLS) to determine the monodispersion, the hydrodynamic diameter and the ζ-potential, and nanoparticle tracking analysis (NTA) to count the NP number, also determining the hydrodynamic diameter. Considering the core dimension, the preferred technique to evaluate the size and the crystallinity is the transmission electron microscopy (TEM). The total amount of metal content can be evaluated by exploiting the use of inductively coupled plasma optical emission spectroscopy (ICP-OES). The surface coating can be assessed by exploiting ^1^H-NMR of the colloidal solution or after the removal of the organic shell from the metal surface. Recently, a great advantage in the determination of the amount of carbohydrate residues loaded on the gold surface has been obtained by using high-performance anion exchange chromatography with pulsed amperometric detection (HPAEC-PAD) [30].

Biodegradable polymeric nanoparticles are another class of suitable nanocarrier for glycans, offering the opportunity to encapsulated payloads, high biocompatibility, surface functionalization capabilities and adjuvanticity [31].

In particular, poly(lactic-coglycolic acid) (PLGA) and poly(lactic acid) (PLA) are largely exploited in biomedicine and have been licensed and approved for human use by US FDA. PLA/PLGA NPs have been envisaged as promising carriers for vaccine delivery, reducing the number of repeated administrations due to the slow antigen release. One benefit of such NPs is that they allow both the absorption of target molecule on the surface and dispersion through the matrix, encapsulating and protecting hydrophobic compounds. PLGA and PLA are generally synthesized by slowly adding the hydrophobic polymers dissolved in an organic phase into an aqueous phase. Nanoparticles immediately form, affording reproducible and monodispersed NPs. This one-step procedure is one of the most diffused and is especially employed when the drug to be added on the NPs is soluble in a volatile, water-miscible solvent. Other manufacturing processes have also been designed, such as the salting-out procedure or the oil-in-water emulsion: in both cases the use of surfactants is necessary. The typical characterization performed to define PLGA and PLA NP size and morphology include the use of Scanning Electron Microscopy (SEM), TEM, atomic-force microscopy (AFM), and DLS [32, 33]. The measurement of ζ-potential is crucial to determine the colloidal stability, while the Fourier transformed infrared (FT-IR) can be exploited to characterize the chemical groups present within the NPs. A micro-BCA assay is employed to determine eventual protein antigen content while an optimized high-throughput version of a phenol–sulfuric acid assay can be used to determine the amount of sugar attached on the PLGA/ PLA-NPs [34]. In particular, a colorimetric method based on reaction of alkaline hydroxylamine reagent with O-acetyl group can be exploited for the evaluation of Vi antigen loading [35].

Liposomes are self-assembled vesicular structures composed of (phospho)lipid bilayers. The general synthesis is based on the sonication of selected phospholipids and cholesterol: the resulting vesicles are versatile, biocompatible and characterized by a low immunogenicity and toxicity [36]. They attract a lot of interest as nanocarriers able to carry multicopy of ligands (coated on their surface, encapsulated or associated within the bilayers) [37]. The design of targeted liposomes involves the coupling of ligands to the surface of preformed vesicles that carry functionalized (phospho)lipid anchors conveniently added in the synthetic mixture [38, 39]. Amphiphilic adjuvants, like lipopeptide TLR ligands, can be associated to carbohydrate antigens in the preparation of the liposomes, to increase immunogenicity and trigger humoral responses [40]. The size of the liposomes that range from 70 to 150 nm can be determined by dynamic light scattering, while the coated-carbohydrate content can be quantified by using the resorcinol-sulfuric acid method (Monsigny’s method) or by exploiting high-performance anion exchange chromatography with pulsed amperometric detection (HPAEC-PAD).

Outer membrane vesicles (OMVs), with a size comprised in the range of 10–300 nm, are a class of bilayer particles discovered in 1982 and naturally released from the outer membrane (OM) of Gram-negative bacteria. OMVs play a crucial role in many biological events and are composed of phospholipids, lipopolysaccharides or lipooligosaccharides, outer membrane proteins, and signaling molecules. In fact, they mimic the bacterial envelope, with the corresponding antigens in native conformations and optimal orientation for the interaction with immune cells. Importantly, OMVs are characterized by immunogenic properties and have been selected as optimal vesicles to develop vaccines against meningococcal pathogens (i.e., MenBvac®, MeNZB®, and BexSero®, VA-MENGOC-BC®) while other OMV-based vaccine candidates are currently under evaluation [41–44].

Recently, to enhance the level of particle production and reduce the reactogenicity while maintaining immunogenicity, generalized modules of membrane antigens (GMMA) have been successfully developed from genetically modified bacteria, manipulated to enhance the in vitro blebbing. Like OMVs, GMMA are spherical vesicles characterized by a variable diameter depending on the bacterial parent strain. Currently, the technology has been successfully applied to various pathogens such as *Salmonella*, *Neisseria meningitidis*, and *Shigella* serotypes [45–47]. GMMA are highly immunogenic, and they have been demonstrated able to confer levels of immunogenicity comparable to the ones of glycoconjugate vaccines. GMMA is a low-cost vesicles-based technology which is considered attractive to produce large quantities of effective vaccines against bacterial diseases.

The size distribution of OMVs and GMMA can be detected by dynamic light scattering and negative staining transmission electron microscopy (TEM) while the O-antigen amount can be determined by HPAEC-PAD.

## Bacterial microbes expressing saccharide antigens for nanosystem development

### Group a *Streptococcus* (GAS)

The gram-positive *Streptococcus pyogenes* or Group A Streptococcus (GAS) is emerging worldwide as a concerning threat. It is the common bacterial cause of sore throats, but can also induce life-threatening infections such as flesh-eating disease, acute rheumatic fever, rheumatic heart disease and streptococcal toxic shock syndrome [48]. GAS is not resistant to penicillin or amoxicillin, the first-line antibiotics to treat the non-severe upper-respiratory infections. However, the use of macrolide antibiotics is required for people who are allergic to penicillin, and the number of estimated invasive GAS infections that are resistant to erythromycin has nearly tripled from 1300 in 2013 to 5400 in 2017 [8]. Rising resistance to antibiotics used in disease treatment is an increasing worldwide concern, especially in less developed regions where higher prevalence rates of invasive GAS are observed [48, 49]. Currently, a safe and effective GAS vaccine is not available. The main component of GAS cell wall is the Lancefield group A carbohydrate (GAC), which is presumably covalently linked to N-acetylmuramic acid (MurNAc) of peptidoglycan. All serotypes of GAS express GAC, which is comprised of a polyrhamnose backbone with *N*-acetylglucosamine (GlcNAc) side chains. The immunodominant GlcNAc epitope of GAC is the basis of all rapid diagnostic testing for GAS infections [50]. GAC has been identified as immunogen for protection against GAS infections. Purified anti-GAC antibodies successfully opsonized different tested GAS serotypes, and mice immunization with GAC induced protection against both intraperitoneal and intranasal GAS challenge [51]. Since rhamnose is absent in mammalian cells, GAC is an attractive candidate for a universal GAS vaccine. Purified GAS-polysaccharide has been conjugated to tetanus toxoid protein and proved able to elicit a protective immune response in a mouse challenge [52]. In addition, it was also demonstrated the immunogenicity of a set of GAS PS fragments, obtained by chemical synthesis. The hexasaccharide fragment, conjugated to CRM197, when tested in mice, conferred levels of immunoprotection comparable to those elicited by the native purified GAS-PS conjugated to CRM197 [53]. It was also revealed that the GAC GlcNAc side chain does not appear as a universal prerequisite for GAS virulence, so that synthetic fragments containing only the polyrhamnose backbone show promising immune-activity [54, 50]. In addition, GlcNAc deficient structures would appear attractive because GlcNAc is also a component of host N-glycosylated proteins, and can mediate immune cross-reactions with human tissue antigens. Indeed, antibodies against GAC may contribute to autoimmunity and to different manifestation of acute rheumatoid fever in a variety of host tissues [55–57]. The case of GAC carbohydrate is an interesting example highlighting the potential of developing simplified structures compared to the natural one, still endowed with immunological properties and more amenable for chemical synthesis. This has prompted a study aimed at highlighting the potential of Au nanoparticles as nanoplatforms for the future development of a GAS vaccine [30]. Tetra- and hexa-rhamnosides have been coated to AuNPs with different morphologies (spherical and urchinated) to exploit the impact of nanoparticle shapes on the resulting saccharide loading and presentation to the antibody. The star-shaped nanoparticles are characterized by a lower antigen density with respect to the spherical ones. In-vitro assays showed that the shape of the NPs does not affect the affinity to the anti-Rha polyclonal antibody, since the affinity of the hexa-rhamnose grafted AuNPs for the antibody is not influenced by the antigen loading and nanoparticle morphology. However, further investigations in-vivo are necessary to address the relationships between NP shapes and their activity, as previous studies reported that NP morphology impacts on biological properties and biodistribution [58].

#### *Mycobacterium tuberculosis*

*Mycobacterium tuberculosis*, the causative agent of tuberculosis (TB), is constantly monitored by Public Health Surveillance System, and classified as a serious threat. The danger is associated to the drug-resistant (DR) forms of the infection that can turn into life-threatening multidrug-resistant (MDR), and extensively drug-resistant (XDR) types. Despite the number of drug-resistant TB cases in the United States has remained stable in the last few years, the necessity of novel strategies for the prevention and treatment of TB is required to reduce the toxicity of the antibiotics used for the most severe forms of the infection, which can cause severe side effects, like hearing loss [59, 60, 61]. In the search for alternative strategies, the cell wall associated lipoglycan lipoarabinomannan (LAM) has been recognized as an attractive target for the development of carbohydrate-based anti-TB vaccines. Vaccination with LAM, and in particular its terminal arabinomannan portion covalently conjugated to mycobacterial protein antigens or TT, offers good protective efficacy in mice and guinea pigs in terms of prolonged survival and reduced pathology [62, 63]. A recent study has highlighted the potential of gold nanoparticles as alternative carriers for glyco-epitopes in anti-TB protein-free vaccine development [64]. Two synthetic terminal-branched hexaarabinofuranoside fragments (Ara6), differing in the length and hydrophobicity of the aglycone linker, were conjugated to 15 nm pre-formed citrate-stabilized AuNPs. Rabbit immunization with the two set of GNPs showed that both antisera contain high titers of antibodies specific for *Mycobacteria*. This study confirms Ara6 epitope as a general mycobacterial antigenic determinant able to induce the production of antibodies specific for cells of different mycobacterial cultures, i.e. *M. bovis*, *M. smegmatis* and *M. phlei*. Furthermore, a new approach for the preparation of glycan-coated GNPs has been reported for the first time. In fact, GNPs were obtained through the use of amino-ending glycosides instead of the common thiol-ending. The Ara6-decorated amino-capped GNPs were stable at basic pH and no aggregation was observed. The use of amino-terminated ligands can affect the efficacy of antibody generation, which is known to be inversely correlated with the stability of the glycan–GNPs conjugates. GNPs are in fact initially phagocytized into phagocytic cells, then the substitution of glycans on the surface of glyco-GNPs with endogenous cellular thiols and amines facilitates the subsequent presentation of saccharide antigen to the surface of macrophages, for B-cells activation. Due to the lower affinity of nitrogen for Au, with respect to sulfur, this mechanism can be facilitated by the use of amino-capped GNPs, but further studies to support this hypothesis are needed.

#### *Neisseria meningitidis*

*Neisseria meningitidis* is together with *Haemophilus influenzae* and *Streptococcus pneumoniae* responsible for most cases of meningitis, in particular those occurring in children, adolescents and young adults. It is the only bacterial species capable of generating epidemics of meningitis. Five serogroups (A, B, C, Y, and W135) are responsible for most of the infections, the majority of cases in Europe and America associated to Groups B and C. On the contrary, group A is the causative agent of cyclic devastating epidemics in Africa [65]. Meningococcal conjugate vaccines, based on CPS of serotypes A, C, W-135, are commercially available and have contributed to provide immunity to general population and reducing large epidemics in African countries [66]. However, they present major drawbacks such as the need for booster doses to maintain protection and the necessity to sustain the so-called “cold-chain” to preserve their activity [67].

D’Souza and Zughaier reported a novel meningococcal nanoparticulate formulation to overcome some of the problems associated to existing vaccines. It consists of meningococcal CPS polymers encapsulated in albumin-based inexpensive biodegradable nanoparticles, that can be stored as a dry powder without degradation facilitating distribution. These CPS-loaded NPs slowly release the antigen and induce a robust innate immune response in in-vitro experiments, and the expression of co-stimulatory molecules necessary for effective antigen presentation by antigen presenting cells [68, 69]. The effect of the addition of adjuvants encapsulated in the meningococcal CPS nanoparticulate (NP) was also assessed, revealing that Alum and MF59 were able to enhance dendritic cell maturation and the expression of antigen presentation markers [70]. A higher stability of the vaccine formulations can also be guaranteed by the development of synthetic vaccines that use modified saccharide fragments, obtained by chemical synthesis [71], endowed with increased chemical stability. In this framework, Men A polysaccharide fragment analogues, in which the phosphodiester bridge connecting the glycosidic units is substituted with a phosphonate as a hydrolytically stable moiety, were developed and assembled on AuNPs to ensure an efficient multipresentation [72, 73]. The AuNPs showed activity in-vitro, which was dependent on the size of the NPs. Furthermore, they were able to induce T cell proliferation as long as a particular length of the saccharide fragments is ensured, at least a disaccharide. In-vivo studies aimed at exploiting these systems as synthetic antigens for immunostimulation are under way. A similar approach, based on hydrolytically stable carba-analogue of MenA and iron oxide nanoparticles (SPIONs) as multivalent platform has also been reported, but in-vitro data are not yet available [74].

A recent study reports a new application of the GMMA technology in the development of affordable vaccines targeting simultaneously different microbes. GMMA vesicles were used as carriers of polysaccharides. In particular, Men A and Men C oligosaccharides were conjugated to GMMA derived from MenB or *S. Typhimurium*. Immunizations with saccharide-GMMA conjugates induced anti-saccharide-specific antibody titers comparable to the corresponding CRM_197_ glycoconjugates, and a superior functional response. Furthermore, the chemical conjugation of types of antigens derived from different pathogens, does not affect the immune response to GMMA, demonstrating the versatility of this platform that can play a dual role of antigen and carrier [75].

### *Pseudomonas aureginosa*

*Pseudomonas aureginosa* is a Gram negative bacterium which is associated to a high virulence factors (i.e.*,* mainly exotoxins and proteases release). It represents one of the leading causes of nosocomial infections and, it is responsible of severe and chronic lung infections in immunocompromised patients [76, 77].

The high rate of morbidity and mortality associated with *P. aureginosa* infections and the development of multi-drug resistance strains [78] are worldwide healthcare issues that have spurred intense research efforts aimed at the identification of new and more effective therapeutic approaches [79, 80]. In this regard, the formation of *biofilms*, which enhances the ability of *P. aureginosa* to resist to antibiotics and to develop infection in harsh media, and the carbohydrate composition of the pathogen surface components (i.e., LPS antigens) offered new opportunities for the identification of therapeutic alternatives to antibiotics.

Hence, the prevention of biofilm formation has been targeted as a strategy to increase *P.aureginosa* susceptibility to antibiotic treatments and it resulted in the development of quorum sensing and efflux pump inhibitors, iron chelators, carbohydrate-based lectin inhibitors [77, 81–83]. Specifically, for the latter, nanoconstructs able to provide the multivalent presentation of glycomimetics/analogues of glycans expressed on the host cells and involved in *P. aureginosa’* lectins binding (*i.e*, LecA and LecB) have been widely investigated [82, 84]. They have been proposed as antiadhesive agents and proved to significantly affect *biofilm* formation by interfering with carbohydrate-lectin interactions at the host-pathogen interface. However, this topic is out of the scope of this review and we refer the reader to more specific reports for further details [82, 84, 85, 86].

No vaccines against *P. aureginosa* are available to date in the market and despite this research field is quite intensive [87, 88], at the best of our knowledge, glycovaccines are mainly based on LPS O-antigens [89]. In this framework, Shafieeardestani and co-workers recently reported on the synthesis and immunogical evaluation of a nanoconjugate that consisted on LPS derivatives extracted from PAO1 strain of *P. aureginosa* entrapped on biocompatible copolymer-nanoparticles based on a mixture of lactic and glycolic acid (PLGA-NPs) [90]. Specifically, detoxified LPS have been conjugated to PLGA nanoparticles by means of EDAC/NHS coupling strategy. Subsequently, the efficacy of the nanoconjugate was assessed either in vitro and in vivo. Data showed that the nanoconjugate was able to elicit IgG antibodies (i.e., IgG1, IgG3, IgG2a and IgG2b) and high levels of cytokines (TNF-a, IFN-γ, and IL-4).

### *Salmonella enterica* serovar Typhi and non-typhoidal *Salmonella*

*Salmonellae* are Gram-negative, facultative anaerobic bacilli. They possess three major antigenic determinants: H or flagellar antigen, O or somatic antigen, and the Vi antigen. The somatic O-antigens are determined by specific glycan sequences on the surface of the cell outer membrane. The Vi capsular antigen is an additional surface antigen, overlying the O-antigen, presented by only a few serovars [91].

One of the strains that express the Vi antigen is *Salmonella* Typhi, the etiologic agent of typhoid fever [92, 93], which is a potentially life-threatening disease leading to bowel rupture, shock and death. Inadequate sanitation conditions and the lack of safe drinking water favors *Salmonella* Typhi infections. Typhoid vaccines, based on the Vi polysaccharide and its fragments are in use and provide good immunity, but a non-complete coverage. Recently, the WHO has recommended the use of the Typhbar TCV® vaccine, where the Vi antigen is conjugated to tetanus toxoid. This vaccine is still not-ideal, but is preferred over the unconjugated Vi polysaccharide (ViPS) and Ty21a vaccines because appropriate for all age groups. Unfortunately, travelling to those country where the disease is common facilitate typhoid fever infections, which require treatment with antibiotics. In the last two decades, several strains of *S.* Typhi have developed resistance to antibiotics. The requirement for alternative preventing strategies to contain the burden of the disease, which is particularly significant in South Asia [94], has stimulated the design of vaccines based on polymer particles. Panda and co. has explored the use of polylactide (PLA) nanoparticles entrapping the Vi-PS as a new vaccine delivery system [35]. These biodegradable polymer nanosystems mimic the natural vaccination, providing a multivalent display of the polysaccharide antigen. Immunization experiments showed that entrapment of Vi antigens in PLA particles improved anti-Vi IgG responses and promoted isotype switching and induction of polysaccharide-specific memory antibody response in mice in a single point immunization. The immunogenicity of the particulate system resulted higher than the one of the soluble Vi antigen. The stronger antibody response is supposed to be generated thanks to multiple factors: multivalent display of Vi polysaccharide on polymer particles; high uptake of PLA-NPs by murine macrophages and dendritic cells with subsequent higher delivery of antigens to antigen presenting cells; sustained release of the antigen from the NPs, which promotes a prolonged presence of the antigen in the lymph nodes and stimulates efficacious antibody response and isotype switching. Overall, the novel polymer-based particle formulations improve the immunogenicity of the Vi T-independent saccharide antigen. The co-entrapment of Vi-PS and a carrier protein (tetanus toxoid) in same polymer particle did not improve the anti-polysaccharide antibody response. More recently, Panda reported an evolution of the former study based on the same rationale. The delivery of a conjugate of Vi-PS with r-flagellin, a TLR5 agonist of *Salmonella* typhi, entrapped in PLA nanoparticles, elicited the generation of antibodies that showed better opsonization and clearance of Salmonella typhi in THP-1 macrophages as compared to Vi-flagellin glycoconjugate and Vi TT (Typhbar®) [33]. Indeed, PLA based particles appear as interesting delivery system which can enhance the immunogenicity of saccharide antigens or weakly immunogenic glycoconjugate vaccines setting the stage for the use of nanotechnology in the design of T cell specific polysaccharide vaccines.

In the framework of the development of controlled release systems, nanoparticles using polymers of chitosan modified by poly (methacrylic acid) (CS–PMAA) have also been exploited to absorb the Vi antigen [95]. The adsorption kinetics and the analytical analyses showed that the Vi antigen can be efficiently loaded into the nanoparticles laying the foundation for a new matrix system for saccharide antigens delivery, but immunological studies are still not available.

*Salmonella enterica* serovars Typhimurium and Enteritidis are responsible for the great majority of invasive nontyphoidal *Salmonella* (iNTS) disease. These infections can evolve into dangerous bloodstream infections, which are a leading cause of death and morbidity in developing countries [96]. Non-typhoidal *Salmonella* is the most common foodborne pathogens causing morbidity, mortality, and burden of disease in all regions of the world. The growing frequency of antimicrobial resistance and the delay in the diagnosis are reducing the effectiveness of antibiotic treatment. Due to the lack of the Vi-CPS, vaccine development is based on different saccharide antigens, i.e. the serovar-specific O-antigen (O-Ag) moiety of Salmonella lipopolysaccharide. Conjugates of different serovars O-Ags to the carrier proteins are immunogenic and protective in mice models [97]. Because of the high incidence of the disease in developing countries and to the high cost of production of conjugated vaccines, the GMMA technology has been considered as an alternative strategy to deliver *Salmonella* lipopolysaccharide O-antigen to the immune system [98]. GMMA outer membrane vesicles, rich in outer membrane antigens, including OAg, were produced from S. Typhimurium and S. Enteritidis strains and used for immunization. Their activity was compared to the one induced by the corresponding O-antigens extracted from wild type bacteria and conjugated to CRM_197_ carrier protein. Immunization with GMMA induced higher anti-O-antigen IgG titers than glycoconjugate administered without adjuvant, while the number was similar in the presence of the adjuvant. GMMA vesicles induced a broader antibody isotype profile, strong bactericidal activity, and resulted more effective than the glycoconjugate in reducing the bacterial colonization after successive infection. Indeed, GMMA platforms offer a promising strategy for the development of nontyphoidal *Salmonella* multivalent vaccines covering more than one-serotype, particularly attractive for low- and middle-income countries due to the simplicity and low-cost of the production process.

#### *Streptococcus pneumoniae*

Pneumonia, a serious respiratory infection mainly caused by *Streptococcus pneumoniae* (SP) bacterium, is able to kill more patients than any other disease. Infected people belong mainly to high-risk group population, i.e. children, elderly and immunocompromised individuals. A recent analysis by UNICEF reports about 2200 cases per day in children under the age of five dying from invasive pneumococcal diseases (IPDs). In other words, pneumonia kills one child every 39 s [99]. SP is an encapsulated bacterium, covered by a dense layer of capsular polysaccharides (CPSs), which represent the main virulence factors. 97 different serotypes have been identified on the basis of the CPS chemical structure [100]. *SP* is non-susceptible to penicillin and to many of the other antimicrobial treatments [8]. The most effective approach to reduce the burden of IPDs and multidrug resistance is vaccination. Pneumonia is in fact considered a preventable disease, thanks to the introduction of pneumococcal vaccines. As already outlined in the Introduction, different generations of pneumococcal vaccines have been developed. Pneumococcal vaccines contain long-chain CPS fragments of the serotypes most frequently associated with IPDs. To date four pneumo vaccines are available on the market, namely the 23-valent polysaccharide-based vaccine Pneumovax23, used mainly for adults, and three conjugate vaccines, Prevnar7, Prevnar10 and Synflorix, covering 7, 10 and 13 serotypes, respectively, able to elicit a more effective protection also in high-risk groups [101, 13]. At the same time, as protection is largely limited to the specific vaccine serotypes, the continuous need of broader coverage to control the outbreak of new emerging serotypes is pushing the development of new vaccines. For example, the 15-valent Merck V114 and the Pfizer’s 20-valent PVC are under evaluation or clinical development and include new serotypes that are associated with IPDs worldwide and are not present in currently licensed vaccines [102–105]. Despite the benefits given by the introduction of PCV vaccines, there are still some drawbacks associated to their production and use (batch-to-batch heterogeneity, microbial contaminations, high cost and long time for development/production/commercialization) that keep the field of anti-pneumococcal vaccine development very active. New technological approaches in pneumo vaccine design rely on the development of multivalent CPS-based conjugate vaccines with flexible composition, which can be constantly modified based on different and changing epidemiological conditions. Another significant advantage can be offered by the chemical synthesis of the saccharide antigens [106–109], which requires the preliminary identification of the minimal CPS structure able to induce a protective immune response. Indeed, the production of saccharide fragments through chemical synthesis is the key to obtain homogeneous and reproducible batches with high level of purity and safety. In this context, nanomaterials play an essential role, since they offer the possibility to modulate the number and the type of ligands loaded on the nanoparticle, providing the possibility to anchor more than one serotype (bi-or multi-antigenic loading) in a multivalent display.

In this context, Pènades group reported in 2012 the first example of the use of gold glyconanoparticles as platforms for the development of fully synthetic pneumococcal vaccines [110]. A branched tetrasaccharide, corresponding to the repeating unit (ru) of *SP* 14 was used as the saccharide minimal epitope able to elicit protective antibodies against SP 14. Hybrid gold nanoparticles (AuNPs) bearing the synthetic single ru of SP14, d-glucose, and OVA_323–339_ peptide as a T-helper moiety in variable molar ratio were prepared. Mice immunization showed that the AuNPs were able to induce the production of specific anti-SP14 IgG antibodies, given the presence of the T-helper peptide on the AuNP. The functionality of the antibodies was positively evaluated through opsonophagocytosis. The IgG titer and opsonophagocytic activity were lower than the ones obtained with the positive control (SP14-CRM_197_ glycoconjugate), but this study represents a pioneering and significant example in the field of nanoparticle based vaccine development.

More recently, results building on the data presented above have been reported [111]. Short synthetic CPS fragments belonging to two different SP serotypes, SP14 and SP19F, were combined together with an immunogenic peptide on the same nanoparticle. In vivo immunization demonstrated an unexpected improvement of immunogenicity against the native polysaccharide type 14 after immunization with the bi-antigenic AuNPs, bearing both types of oligosaccharide epitopes. Furthermore, the presence of SP19F together with SP14 repeating unit on the same nanoparticle triggered an anti-SP14 immune response comparable with commercially available PCV13 vaccine.

Glycoengineered Outer Membrane Vesicles (geOMVs) were assessed as a versatile technology for the design of new pneumococcal vaccine. Price et al. developed a geOMV platform using an *E. coli* strain engineered to produce OMVs carrying SP 14 CPS on their surface [41]. Sera of mice injected with SP14-OMVs cross-react with SP 14 CPS. IgG titers and efficacy in OPA tests were similar to the one induced by mice immunization with commercial Prevnar 13. This technology can be extended to the preparation of geOMVs expressing bacterial surface polysaccharides encoded by other bacterial types. Hence, the low-cost geOMV technology offers the advantage to develop in the future a multivalent vaccine generated by mixing geOMVs carrying capsules from different serotypes.

Anish et al. developed polylactide micro- and nano-particle systems entrapping the capsular polysaccharide of SP serotype-1, and found that IgG titers significantly depend on the size of the particle [32]. The delivery of the saccharide antigen in polymeric particles enhances its immunogenicity. NPs, more than micro systems, allow the antigen to be presented with an efficient surface organization and epitope density, which are key factors for generating robust IgG responses. The delivery of pneumococcal proteins (PspA or PsaA) together with SP 1 CPS (PLA particles co-entrapping sugar antigen and T-helper) was not able to improve the anti-polysaccharide response.

### *Shigella*

Shigellosis is a serious intestinal infections caused by *Shigella* species which consist on a large class Gram-negative bacteria that belongs to Enterobacteriaceae family [112, 113]. To date four species including more than 50 serotypes have been identified.

Several approaches have been undertaken to develop a *Shigella* vaccine and they are mainly based on the targeting of the outer polysaccharide antigen (O-antigen) of the lipopolysaccharide (LPS) [114, 115]. However, the main challenge is related to the overcome of the serotype-specificity of the antibody response and thus, the need of broadly protective vaccines. Among synthetic glycoconjugate vaccines, data include studies on *S. flexneri* serotype 2a and *S. dysenteriae* type 1 and they mainly consist on constructs where the specific-O-polysaccharides have been conjugated to immunogenic carrier proteins [116, 115, 117, 118]. In this framework, nanotechnology has been successfully exploited to obtain a liposomes vaccine prototype [39]. In particular, the glycoliposomes consisted on a SF2a synthetic pentasaccharide as O-antigen mimic (B cell epitope) and a Th epitope derived from influenza virus hemagglutinin (HA). Specifically, the two epitopes were conjugated to the liposomes surface thought the maleimide residues of a S-[2,3-bis(palmitoyloxy)-2(R)-propyl]-N-palmitoyl-(R)-cysteinylalanyl-glycine (Pam3CAG) anchor. The fully synthetic liposomes proved effective in eliciting antibody responses against the native LPS in vivo.

In an attempt to overcome serotype-specificity of the antibody response, a vaccine platform based on Generalized Modules for Membrane Antigens (GMMA) derived from a *S. sonnei* strain was proposed [45]. In order to reduce the endotoxicity of GMMA, *S. sonnei* strain was genetically modified to produce a pentaacylated lipopolysaccharide whereas maintaining the virulence plasmid encoding for the O-antigen component of the LPS. The *S. sonnei* GMMA vaccine is now in Phase 1 clinal trial and in vivo data in mice confirmed either the low potential for reactogenicity and a good tolerability by all the routes of administration (i.e., intramuscular, intranasal, and intradermal) evaluated. Worth noting, GMMA vaccine were highly immunogenic in mice and in rabbits eliciting a substantial anti-LPS antibody levels after a single immunization dose.

## Conclusion

Different studies have demonstrated that nanomaterials offer several opportunities to combat bacterial infections. Recently, they have been considered also as carriers for the formulation of alternative antibacterial vaccines. Many of the licensed vaccine against bacterial pathogens are based on saccharide antigens. The opportunity of inducing a specific immune response against a bacterial saccharide has been exploited also in nanosystem formulations. Nanoparticles coated or entrapped with carbohydrate epitopes are able to efficiently stimulate the immune system with key advantages in terms of a sustained release/presentation of the antigen and high immunogenicity of the system exerted by the nature of the nanoparticle. Novel carriers might offer the opportunity to produce safer formulations, overcoming immune interference associated to contaminants at the moment present in licensed vaccines based on CPS obtained by extractive and semipreparative methods. Furthermore, multivalent vaccines carrying both protein and saccharide epitopes of the same pathogen or targeting multibacterial species can be designed.

This methodology requires an in-depth knowledge of the saccharides that are responsible for bacterial virulence. In fact, despite cell surface carbohydrates (glycans) are targets for the development of glycoconjugate nanomaterials [119], there is a number of bacterial species for which this approach has not yet been exploited. This is for example the case of *Acinetobacter baumanii*, one of the major global health threats, which is responsible for antibiotic resistant infections that tend to occur in patients who recently received care in healthcare units [120]. Despite the immunogenicity of K1 capsular polysaccharide of *A. baumannii* has been demonstrated [121], there are still limited data available on the role the other different CPS types play in causing disease, which requires further studies to determine the feasibility of CPS as a target for new strategies to combat this increasingly persistent and deadly human pathogen [122].

In addition, the reported studies highlight that promising nanomaterial based applications can aptly build on the availability of solid synthetic protocols for the obtainment of the saccharide epitope fragments.

In this conceptual framework, the results discussed herein highlight that the progress of new strategies in fighting infection disease can effectively hinge on the development of nanoparticle-based vaccines. Indeed, vaccination strategies are an effective tool to prevent infections in the community, including those that can be resistant. In addition, vaccination contributes to reduce the carriage and the transmission of the infection to unvaccinated individuals, thus slowing the spread of pathogens, and eventually minimizing the chance of evolving antimicrobial resistance.
