# Correction to: Glyconanoparticles as tools to prevent antimicrobial resistance

**DOI:** 10.1007/s10719-021-09995-7

**Published:** 2021-04-02

**Authors:** Laura Morelli, Laura Polito, Barbara Richichi, Federica Compostella

**Affiliations:** 1 Department of Medical Biotechnology and Translational Medicine, University of Milan, Via Saldini 50, 20133 Milan, Italy; 2 National Research Council, CNR-SCITEC, Via G. Fantoli 16/15, 20138 Milan, Italy; 3 Department of Chemistry ‘Ugo Schiff’, University of Florence, Via della Lastruccia 13, 50019 Sesto Fiorentino, FI Italy

**Correction to: Glycoconjugate Journal.**

10.1007/s10719-021-09988-6

The original article has been corrected. The correct article title should be “Glyconanoparticles as tools to prevent antimicrobial resistance” instead of “Glyconanano particles as tools to prevent antimicrobial resistance.

